# Dairy Cattle Density and Temporal Patterns of Human Campylobacteriosis and Cryptosporidiosis in New Zealand

**DOI:** 10.1007/s10393-022-01593-9

**Published:** 2022-06-10

**Authors:** Leah Grout, Jonathan Marshall, Simon Hales, Michael G. Baker, Nigel French

**Affiliations:** 1 Department of Public Health, University of Otago, Wellington, 6021 New Zealand; 2 School of Mathematical and Computational Sciences, Massey University, Palmerston North, 4474 New Zealand; 3 School of Veterinary Science, Hopkirk Research Institute, Massey University, Palmerston North, 4474 New Zealand

**Keywords:** dairy cattle density, zoonoses, public health

## Abstract

Public health risks associated with the intensification of dairy farming are an emerging concern. Dairy cattle are a reservoir for a number of pathogens that can cause human illness. This study examined the spatial distribution of dairy cattle density and explored temporal patterns of human campylobacteriosis and cryptosporidiosis notifications in New Zealand from 1997 to 2015. Maps of dairy cattle density were produced, and temporal patterns of disease rates were assessed for urban versus rural areas and for areas with different dairy cattle densities using descriptive temporal analyses. Campylobacteriosis and cryptosporidiosis rates displayed strong seasonal patterns, with highest rates in spring in rural areas and, for campylobacteriosis, summer in urban areas. Increases in rural cases often preceded increases in urban cases. Furthermore, disease rates in areas with higher dairy cattle densities tended to peak before areas with low densities or no dairy cattle. Infected dairy calves may be a direct or indirect source of campylobacteriosis or cryptosporidiosis infection in humans through environmental or occupational exposure routes, including contact with animals or feces, recreational contact with contaminated waterways, and consumption of untreated drinking water. These results have public health implications for populations living, working, or recreating in proximity to dairy farms.

## Introduction and Purpose

Dairy cattle numbers have increased substantially in New Zealand in recent decades (MacLeod and Moller 2006; Statistics New Zealand 2012). Dairy cattle are a known reservoir for a number of different pathogens that can cause human illness (FAO et al. 2006; Toth et al. 2013; Grout et al. 2020), including *Campylobacter* spp. and *Cryptosporidium* spp. (Cavirani 2008; Toth et al. 2013; Whitfield et al. 2017; Grout et al. 2020). It is possible that increases in dairy cattle numbers and densities could lead to increased exposure to zoonotic pathogens and increased disease rates in humans. However, there have been relatively few studies examining the potential health effects of the intensification of dairy farming in the country.

Campylobacteriosis is the most commonly notified disease in New Zealand (ESR 2017). Case rates appear to be highly seasonal and tend to peak in the spring or summer (Spencer et al. 2012). Potential drivers of seasonality are thought to include increased shedding in animal reservoirs and higher contamination in the food chain, as well as changes in human behaviors (Spencer et al. 2012). In urban areas, notification of campylobacteriosis cases is thought to be driven by the consumption of contaminated food products (Spencer et al. 2012). Specifically, poultry was identified as the primary source of human campylobacteriosis due to *C. jejuni* infection in New Zealand (Mullner et al. 2009). Several interventions were introduced in the poultry industry in late 2006, and by 2008, the annual campylobacteriosis rate had dropped by 54% compared to the average annual rate for the period from 2002 to 2006 (Sears et al. 2011). Despite reductions in the contribution of poultry after intervention it remained the dominant source of human infection, with an estimated 84% of cases in New Zealand infected with strains attributed to a poultry source and 14% infected with strains attributed to cattle (Lake et al. 2021). Environmental exposures, including direct contact with animals, contact with feces, recreational contact with contaminated waterways, and consumption of untreated drinking water, may play a larger role in transmission in rural areas (Spencer et al. 2012).

Cryptosporidiosis notifications tend to peak in the spring (September–November in New Zealand) (Snel et al. 2009; Lal et al. 2016), predominantly in rural areas (Snel et al. 2009). A smaller late summer or early autumn peak is also occasionally seen in urban areas (Snel et al. 2009). Evidence suggests that the spring peak in cryptosporidiosis cases may be due to spring calving and zoonotic or indirect environmental transmission of *Cryptosporidium parvum*, while the autumn peak in urban areas is consistent with anthroponotic transmission of *C. hominis*, often through contaminated swimming pools (Learmonth et al. 2004; Snel et al. 2009).

While the seasonality of these diseases has been widely acknowledged, the drivers of seasonal patterns are not well understood. Social and environmental factors, including climate, land use, and livestock density variables, can interact across spatial and temporal scales to influence disease risk (Lal et al. 2013; Lal 2014; Cherrie et al. 2018). Few studies have examined temporal patterns of disease in urban and rural areas, and even fewer have examined seasonality in areas with different livestock densities (Lal et al. 2012). Establishing temporal trends for zoonotic enteric diseases allows for the identification of potential risk factors (Lal 2014; Chen et al. 2015). Identifying seasonal trends also allows for the monitoring of changes in disease patterns, which is useful for understanding how environmentally sensitive diseases will respond under future scenarios of climate change or land use change (Patz 2002; Lal 2014). Therefore, this study seeks to review and describe spatial trends in dairy cattle density from 2000 to 2014 and to explore the temporal patterns of disease notifications from 1997 to 2015 across New Zealand.

## Methods

### Dairy Cattle Data Collection

#### Dairy Cattle Density

Dairy cattle numbers were obtained from the Agribase™ database for the years 2000, 2006 and 2014 at the meshblock level (AsureQuality 2019). Meshblocks are the smallest geographic unit for which statistical data are collected and processed by Statistics New Zealand (2014). A meshblock is a defined area that varies in size from part of a city block to large areas of rural land (Statistics New Zealand 2014). Dairy cattle numbers for each farm were mapped to a single meshblock according to the location of the main farmgate or to the physical location of the home (i.e., even if a farm spanned across multiple meshblocks, the dairy cattle numbers were assigned to a single meshblock in the database). Dairy cattle density per square kilometer was calculated for each year. Average dairy cattle density was also calculated across the three years for which data were obtained and meshblocks were categorized as areas with no dairy, low dairy density (> 0–13.3 cows/km^2^), medium dairy density (> 13.3–102.3 cows/km^2^), and high dairy density (> 102.3 cows/km^2^). The categories of dairy cattle density were based on tertiles for all meshblocks with a dairy cattle density greater than zero.

#### Change in Dairy Cattle Densities

The change in dairy cattle density was calculated by subtracting the calculated densities for 2000 from the densities for 2014. The change in dairy cattle density was then mapped at both the meshblock and census area unit (CAU) levels. CAUs are the second smallest geographic unit for which statistical data are collected and processed by Statistics New Zealand (2014). A CAU is a defined area that varies in size and is comprised of multiple meshblocks (Statistics New Zealand 2014). The change in dairy cattle density was examined at the CAU level in order to smooth dairy cattle density and partially account for the fact that dairy cattle numbers were allocated to a single meshblock even if a farm spanned multiple meshblocks.

### Human Data Collection

#### Human Case Data

Notified cases of campylobacteriosis and cryptosporidiosis from 1997 to 2015 in New Zealand were obtained from the National Notifiable Disease Surveillance system. No major changes were made to the surveillance of these notifiable diseases from 1997 to 2007, but direct laboratory notification began in 2008 (Ministry of Health 2007). Notified cases were assigned to a meshblock based on the home address of the patient. When the address for a case was unknown, it was geocoded to the address of the regional Public Health Service.

#### Census Data

Meshblock level population estimates were obtained for census years 2001, 2006, and 2013 from Statistics New Zealand. Population estimates were used to calculate disease incidence rates per 100,000 population.

#### Urban/Rural Profile

Statistics New Zealand classifies meshblocks as either (i) main urban areas, (ii) satellite urban areas, (iii) independent urban areas, (iv) rural areas with high urban influence, (v) rural areas with moderate urban influence, (vi) rural areas with low urban influence, (vii) highly rural/remote areas, or (viii) areas outside urban/rural profile. For the purposes of this study, the three categories for urban areas were combined into a single ‘Urban’ category and the four categories for rural areas were combined into a single ‘Rural’ category.

### Assessing the Spatial Distribution of Dairy Cattle Density

First, dairy cattle density was mapped at the meshblock level for the years 2000, 2006, and 2014. These years were chosen as they most closely matched the dates for the census (see 'Census Data' above). Next, the change in dairy cattle density from 2000 to 2014 was mapped at the meshblock and CAU levels. Specifically, the change in dairy cattle density was categorized as those areas with (i) no dairy, (ii) a decrease in dairy density, (iii) no change in dairy cattle density, (iv) a small increase in dairy cattle density, (v) a medium increase in dairy cattle density, and (vi) a large increase in dairy cattle density from 2000 to 2014. Categories of increased dairy cattle density were based on tertiles for all areas with an increase in density. ArcGIS was used to produce all maps (ESRI 2018).

### Assessing Temporal Patterns in Human Campylobacteriosis and Cryptosporidiosis Cases

#### Seasonal Trend Decomposition by Regression

Disease notification data were aggregated to week, month, and year. Then, a seasonal-trend decomposition by regression (STR) was undertaken using disease notification data aggregated at the weekly level to smooth the day-of-the-week effect (i.e., cases are not typically reported on Saturday or Sunday, due to weekend closures). Analyses were conducted using the R package stR (Dokumentov 2018).

#### Monthly Plots

Next, monthly plots were created in order to further examine the difference between monthly incidence rates across urban versus rural areas, and across categories of dairy cattle density (see ‘*Dairy Cattle Density*’ above). However, campylobacteriosis data were restricted to the period 2010–2015 in order to examine seasonal patterns after the implementation of interventions in the poultry industry in 2006. First, the average campylobacteriosis case counts for each month were calculated for the period 2010–2015 and the average cryptosporidiosis case counts for each month were calculated for the period 1997–2015. Then, monthly incidence rates were calculated by dividing the average monthly case counts for campylobacteriosis by the 2013 population, while the monthly case counts of cryptosporidiosis were divided by the population estimate for 2006. The most central census estimates were used for each dataset. The mean monthly incidence rates per 100,000 population were then plotted, with 95% confidence intervals for the means calculated through the default method in SPSS Statistics.

## Results

### Spatial Distribution of Dairy Cattle Density

**Figure 1:**
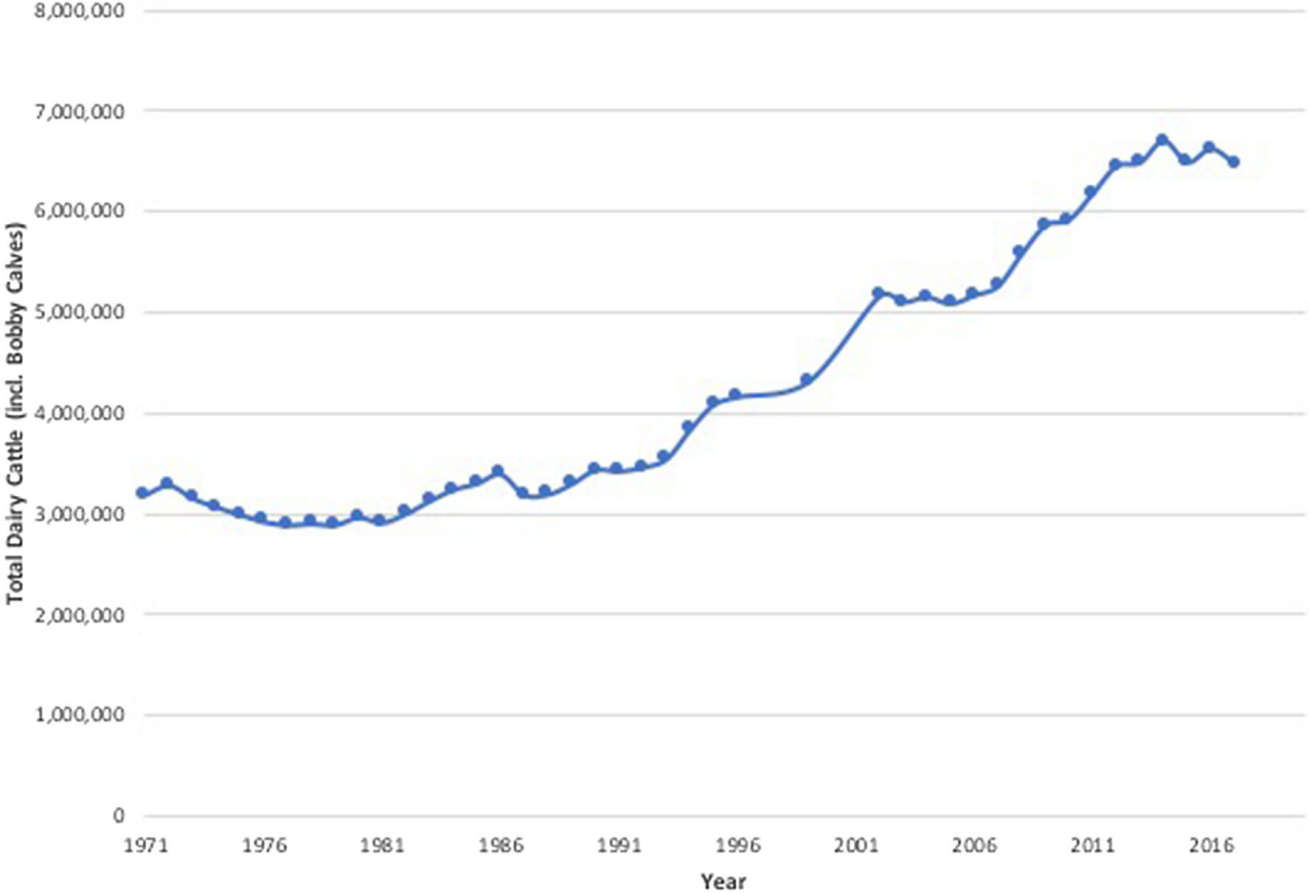
Total dairy cattle in New Zealand (including bobby calves). There was no Agricultural Survey carried out in 1997 or 1998 Adapted from Statistics New Zealand (2017).

**Figure 2:**
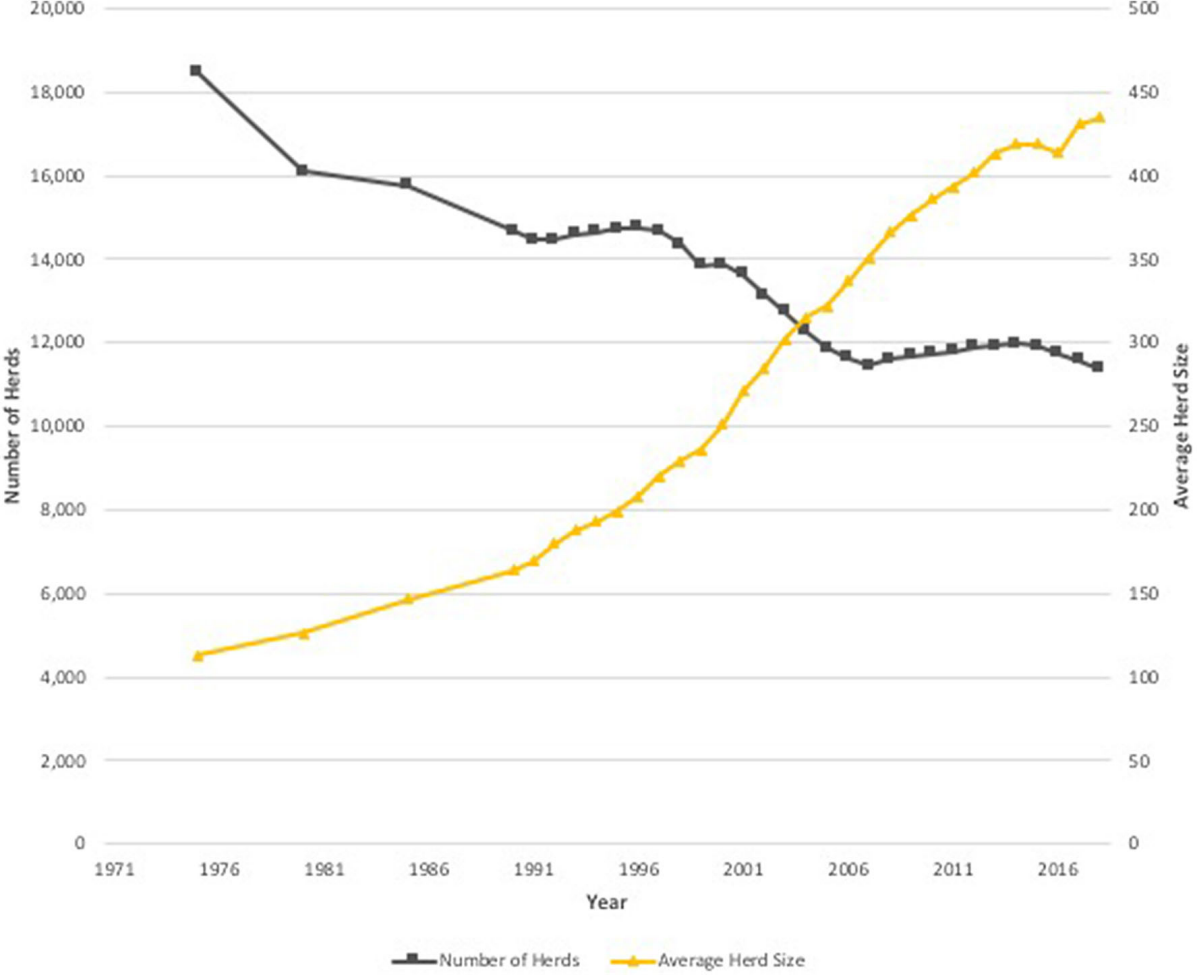
Total number of dairy herds and average herd size in New Zealand Adapted from LIC & DairyNZ (2019).

In New Zealand, many producers have relied on higher stocking rates to increase production; using the same land area to support more cattle has increased livestock density. Stocking rates for dairy cattle have increased rapidly since 1990 (Fig. 1) (MacLeod and Moller 2006), and the number of dairy cattle increased by more than one million from 2007 to 2012 (Statistics New Zealand 2012). Average herd size has also increased, while the number of herds in New Zealand has fallen (Fig. 2) (LIC et al. 2019), and in 2016, there were 11,918 dairy herds and approximately 5 million milking cows in New Zealand (DairyNZ 2016).

**Figure 3:**
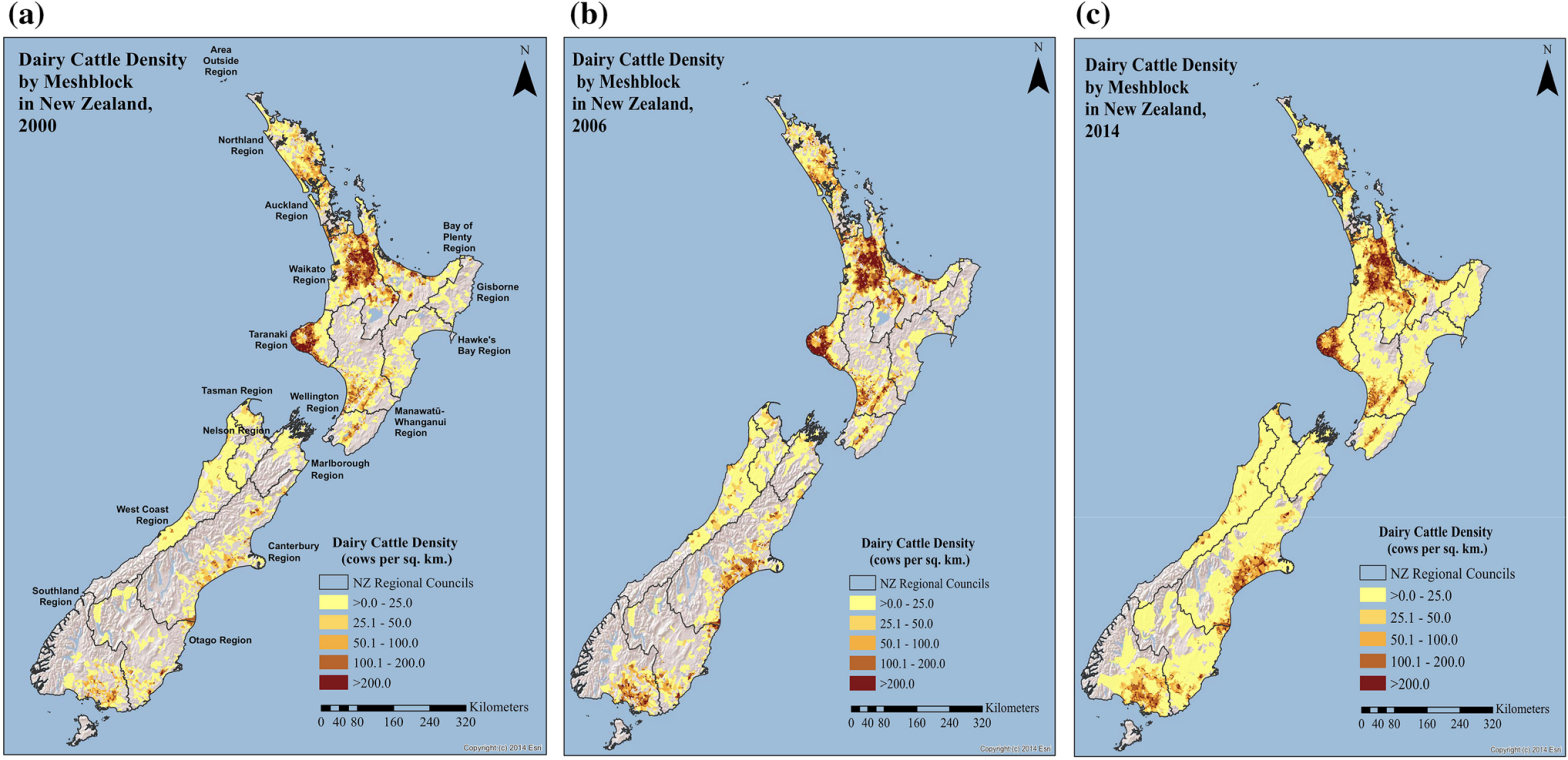
Dairy cattle density in New Zealand in (**a**) 2000, (**b**) 2006, and (**c**) 2014 at the meshblock level. Adapted from Agribase™ database.

Maps of dairy cattle density for 2000, 2006, and 2014 show that in the North Island, the regions that had higher dairy cattle densities in 2000 (i.e., Waikato and Taranaki) maintained higher densities over time (Fig. 3). However, the maps also indicate that dairy cattle were introduced to new regions throughout the North Island from 2000 to 2014.

As in the North Island, dairy cattle density increased in parts of the South Island where once there were no dairy cattle. However, certain areas of the South Island witnessed a very rapid increase in density. Rapid increases in dairy cattle density were particularly evident in the Canterbury Plains and Southland regions.

**Figure 4:**
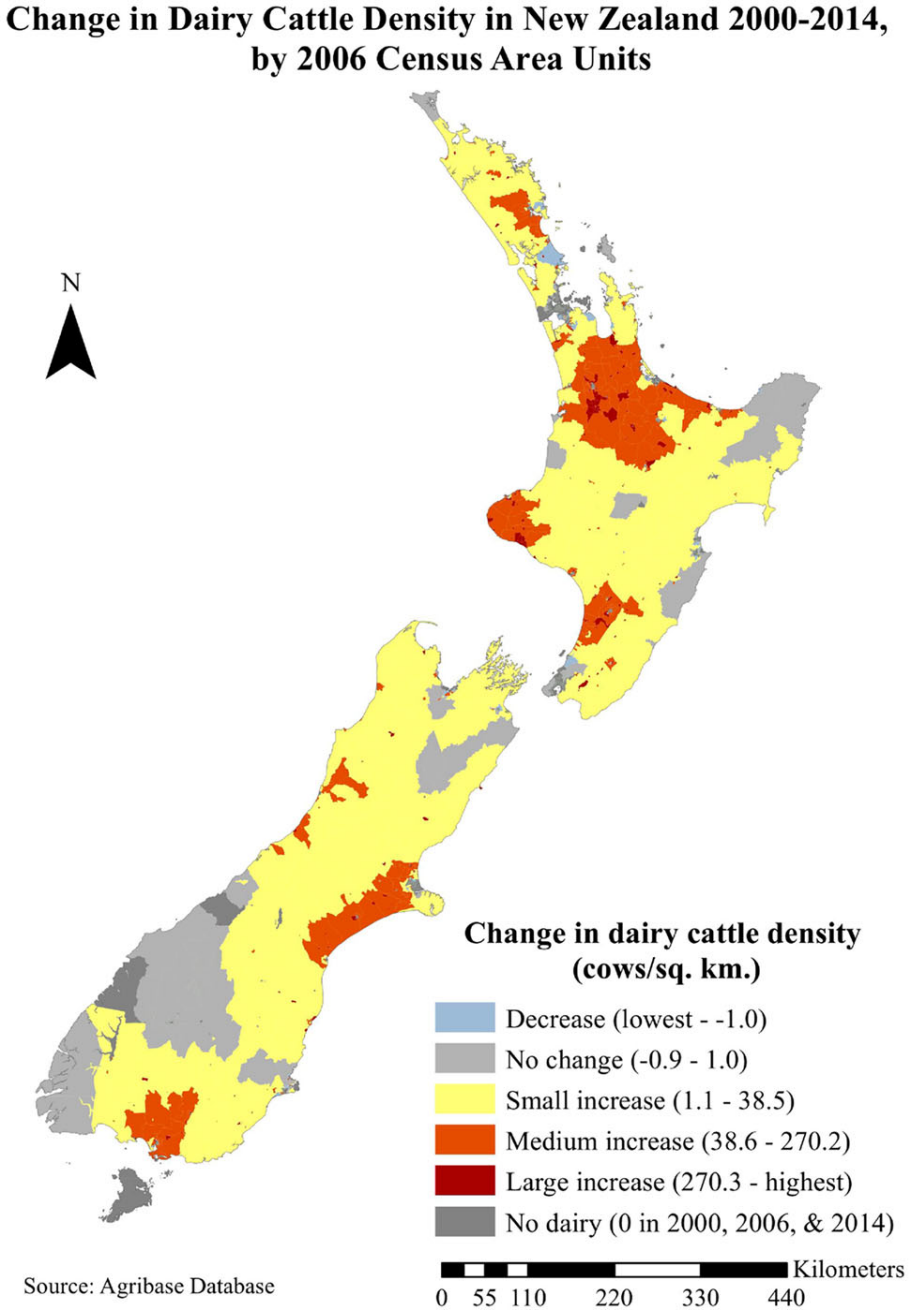
Change in dairy cattle density in New Zealand from 2000 to 2014 at the census area unit level. Adapted from Agribase™ database.

Aggregation to the CAU level helped to highlight regional differences (Fig. 4). In the North Island there were substantial increases in dairy cattle density in the Waikato and Bay of Plenty regions, Taranaki, lower Manawatu-Wanganui, and parts of Northland. In the South Island, there were substantial increases in dairy cattle density in the Canterbury Plains, Southland, and in parts of the West Coast. Smaller increases were seen in many other parts of the country.

### Temporal Patterns in Human Campylobacteriosis and Cryptosporidiosis Cases

#### Seasonal-Trend Decomposition by Regression

**Figure 5:**
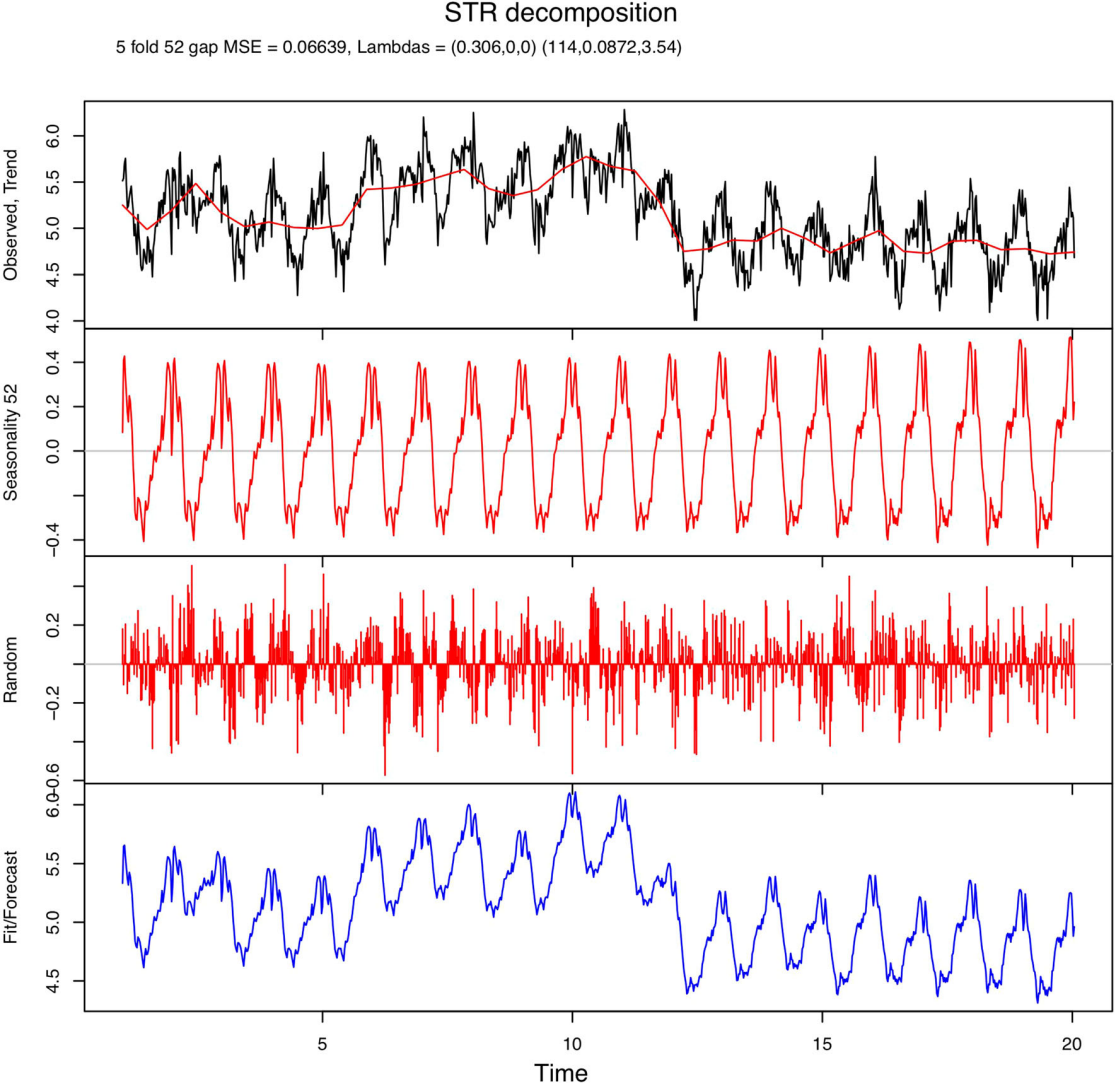
Seasonal decomposition of the weekly incidence of campylobacteriosis in New Zealand (1997–2015). MSE = mean squared error; Lambdas = smoothing parameters; Trend = the long-term temporal trend; Seasonality = a repeating seasonal pattern that changes slowly or remains constant over time; Random = the remainder of the data after the Trend and Seasonality components are removed; Fit/Forecast = Trend + Seasonality. The original data can be obtained by adding the Trend, Seasonality, and Random components.

**Figure 6:**
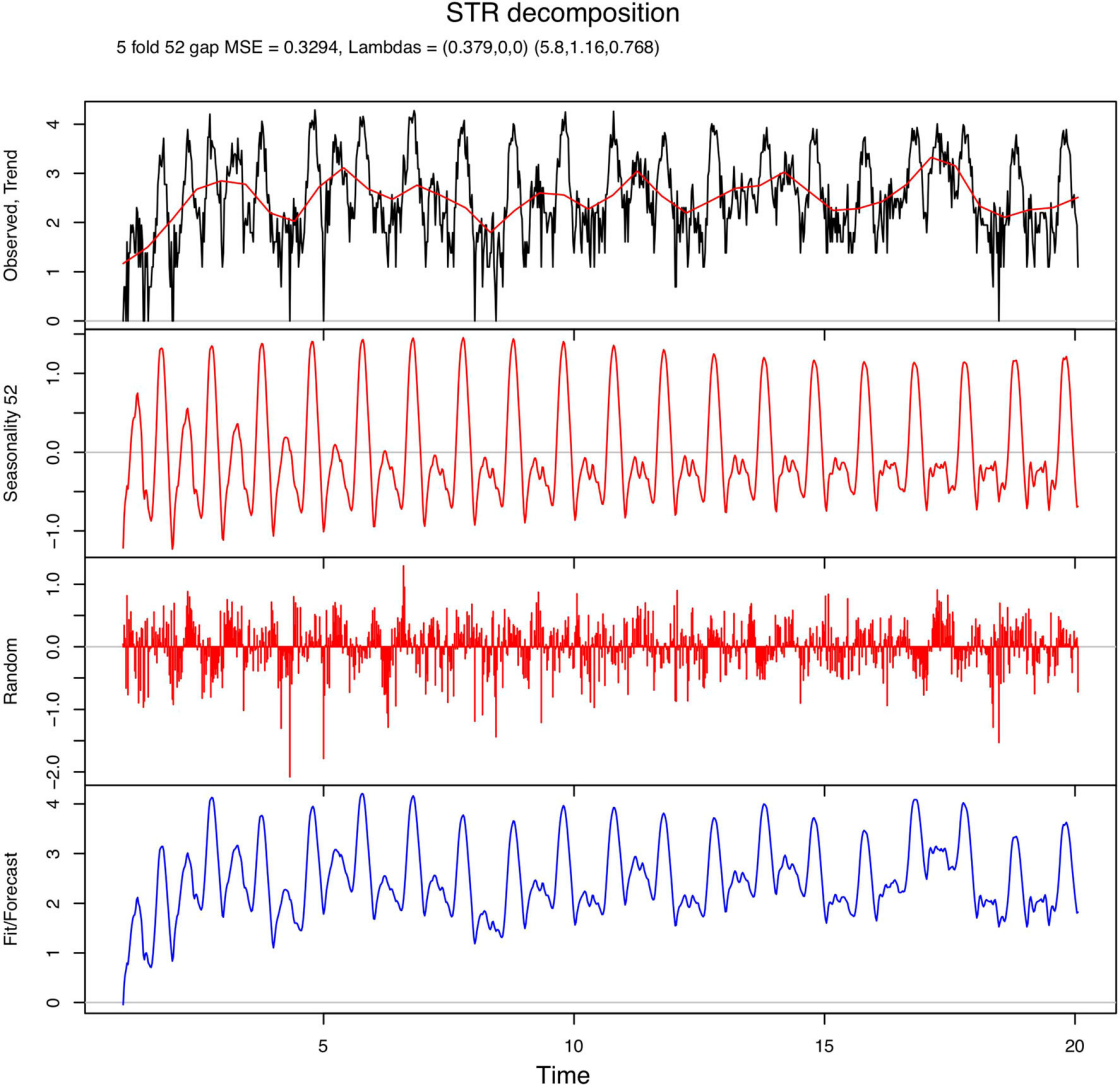
Seasonal decomposition of the weekly incidence of cryptosporidiosis in New Zealand (1997–2015). MSE = mean squared error; Lambdas = smoothing parameters; Trend = the long-term temporal trend; Seasonality = a repeating seasonal pattern that changes slowly or remains constant over time; Random = the remainder of the data after the Trend and Seasonality components are removed; Fit/Forecast = Trend + Seasonality. The original data can be obtained by adding the Trend, Seasonality, and Random components.

The seasonal decomposition of weekly incidence rates of campylobacteriosis showed that the incidence peaked in the summer (Fig. 5), while the trend line displays the decrease in incidence rates following poultry industry interventions that were introduced in 2006. The seasonal decomposition of the incidence of cryptosporidiosis indicated a bi-modal seasonal pattern, with a large peak observed in the spring and a smaller peak observed in the autumn (Fig. 6).

#### Monthly Plots

Monthly plots were created for urban and rural meshblocks in New Zealand to further examine differences in the seasonality of disease notification rates.

**Figure 7:**
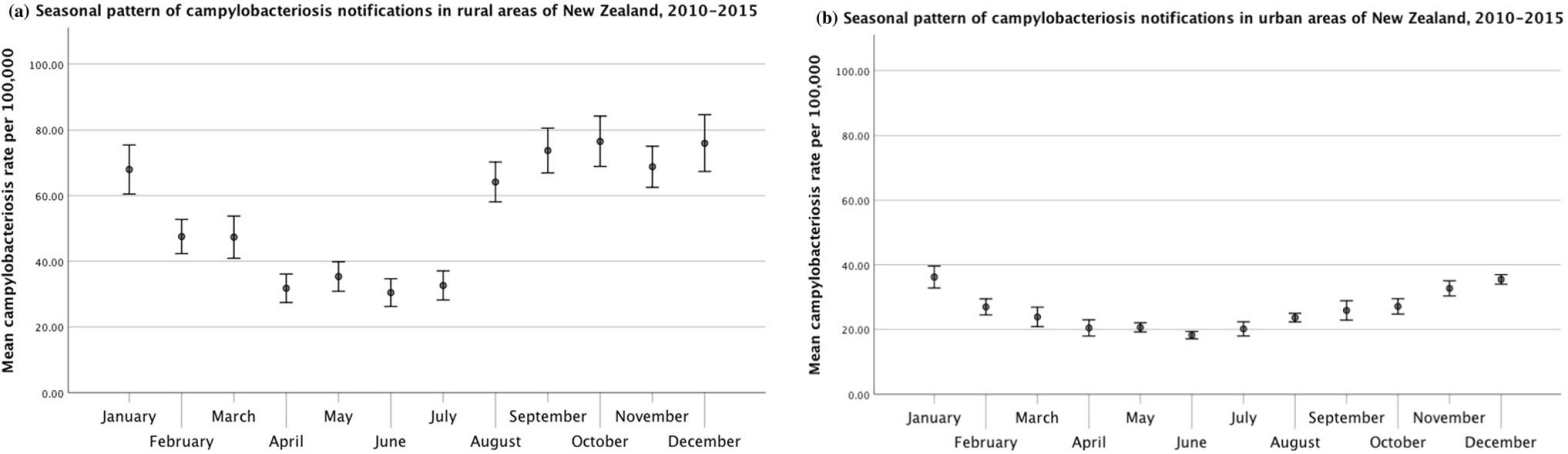
Seasonal pattern of human campylobacteriosis rates stratified by (**a**) rural and (**b**) urban and status. Error bars represent 95% confidence intervals for the means.

For campylobacteriosis, the plots below summarize the average rates for each month from 2010 to 2015. In urban areas, campylobacteriosis rates peaked in the summer (i.e., January), while in rural areas, rates began to increase in August, exhibited a spring peak, and then remained relatively high through most of the summer (Fig. 7, Table 1).

**Table 1:** Average Monthly Campylobacteriosis and Cryptosporidiosis Incidence Rates per 100,000 for Urban Versus Rural Areas, and for Areas with Different Dairy Cattle Densities in New Zealand for the Period 2010–2015 and 1997–2015, Respectively.

	Month	Urban areas	Rural areas	Areas with no dairy cattle	Areas with low dairy cattle density	Areas with medium dairy cattle density	Areas with high dairy cattle density
		Mean incidence rate per 100,000 (95% CI)	Mean incidence rate per 100,000 (95%CI)	Mean incidence rate per 100,000 (95%CI)	Mean incidence rate per 100,000 (95%CI)	Mean incidence rate per 100,000 (95%CI)	Mean incidence rate per 100,000 (95%CI)
Campylobacteriosis (2010–2015)	January	36.23 (32.85 to 39.61)	67.95 (60.48 to 75.42)	37.35 (33.69 to 41.00)	80.38 (66.06 to 94.71)	53.31 (45.78 to 60.84)	42.75 (36.96 to 48.54)
	February	26.98 (24.48 to 29.49)	47.52 (42.30 to 52.74)	28.00 (25.28 to 30.73)	54.89 (46.38 to 63.40)	35.48 (29.76 to 41.20)	31.97 (26.94 to 37.00)
	March	23.87 (20.86 to 26.88)	47.32 (40.91 to 53.74)	24.98 (21.67 to 28.29)	53.92 (43.05 to 64.79)	36.57 (31.12 to 42.01)	28.38 (22.13 to 34.62)
	April	20.45 (17.93 to 22.96)	31.75 (27.42 to 36.09)	20.98 (18.29 to 23.67)	38.52 (30.31 to 46.73)	24.26 (19.88 to 28.65)	21.70 (17.63 to 25.78)
	May	20.62 (19.18 to 22.06)	35.33 (30.83 to 39.83)	21.14 (19.55 to 22.72)	43.86 (35.53 to 52.20)	26.79 (22.46 to 31.12)	22.88 (18.31 to 27.44)
	June	18.21 (17.06 to 19.35)	30.42 (26.20 to 34.66)	18.51 (17.41 to 19.62)	39.67 (29.98 to 49.37)	24.51 (19.95 to 29.06)	17.62 (13.37 to 21.87)
	July	20.15 (17.95 to 22.35)	32.62 (28.19 to 37.05)	20.58 (18.23 to 22.93)	39.08 (30.25 to 47.91)	23.81 (19.80 to 27.81)	24.83 (20.25 to 29.41)
	August	23.64 (22.29 to 24.99)	64.15 (58.08 to 70.21)	23.46 (21.91 to 25.01)	61.16 (51.45 to 70.88)	54.78 (48.31 to 61.25)	56.72 (48.75 to 64.70)
	September	25.88 (22.88 to 28.89)	73.75 (66.93 to 80.57)	25.04 (21.85 to 28.24)	72.44 (60.38 to 84.50)	69.10 (60.68 to 77.52)	61.01 (53.32 to 68.70)
	October	27.14 (24.73 to 29.54)	76.51 (68.88 to 84.14)	27.79 (25.15 to 30.42)	79.17 (64.42 to 93.93)	69.40 (61.51 to 77.30)	46.66 (40.44 to 52.89)
	November	32.73 (30.39 to 35.07)	68.81 (62.54 to 75.08)	32.93 (30.35 to 35.52)	73.65 (63.85 to 83.44)	60.98 (53.78 to 68.18)	49.87 (43.37 to 56.36)
	December	35.48 (34.03 to 36.93)	75.94 (67.35 to 84.53)	36.20 (34.30 to 38.09)	84.21 (68.45 to 99.97)	66.93 (59.09 to 74.77)	47.17 (40.88 to 53.46)
Cryptosporidiosis (1997–2015)	January	4.28 (3.32 to 5.25)	5.64 (3.62 to 7.67)	4.99 (3.85 to 6.12)	8.44 (3.95 to 12.93)	2.55 (1.41 to 3.69)	1.46 (0.70 to 2.21)
	February	4.45 (3.91 to 5.00)	3.75 (2.06 to 5.44)	4.59 (4.00 to 5.17)	4.66 (2.33 to 6.98)	4.35 (1.25 to 7.45)	1.64 (1.00 to 2.27)
	March	7.52 (6.40 to 8.64)	4.06 (2.85 to 5.28)	7.95 (6.73 to 9.17)	6.20 (3.51 to 8.90)	2.75 (1.77 to 3.73)	2.69 (1.56 to 3.83)
	April	6.65 (5.79 to 7.52)	3.23 (2.33 to 4.14)	7.01 (6.07 to 7.96)	4.94 (3.28 to 6.59)	2.03 (1.30 to 2.75)	2.58 (1.42 to 3.74)
	May	6.53 (5.19 to 7.86)	4.66 (2.88 to 6.43)	6.78 (5.34 to 8.21)	5.66 (2.73 to 8.60)	3.43 (1.97 to 4.89)	4.58 (1.10 to 8.06)
	June	3.75 (2.99 to 4.51)	4.00 (2.26 to 5.75)	4.00 (3.17 to 4.82)	4.15 (1.85 to 6.45)	3.43 (0.64 to 6.22)	2.78 (0.74 to 4.82)
	July	3.37 (2.61 to 4.13)	3.56 (2.56 to 4.57)	3.55 (2.73 to 4.37)	3.47 (1.45 to 5.50)	2.81 (1.87 to 3.75)	3.26 (1.75 to 4.78)
	August	5.01 (4.20 to 5.82)	17.64 (14.35 to 20.93)	4.41 (3.59 to 5.22)	14.13 (6.36 to 21.89)	15.14 (12.58 to 17.70)	21.30 (17.05 to 25.56)
	September	9.98 (9.28 to 10.69)	58.36 (50.91 to 65.81)	9.55 (8.62 to 10.48)	49.17 (31.63 to 66.72)	42.72 (37.51 to 47.93)	63.27 (54.82 to 71.71)
	October	15.16 (13.60 to 16.72)	58.60 (50.36 to 66.84)	15.01 (13.55 to 16.47)	67.36 (46.89 to 87.83)	47.35 (40.00 to 54.70)	42.25 (34.12 to 50.37)
	November	7.61 (6.51 to 8.70)	25.69 (21.47 to 29.91)	7.83 (6.68 to 8.97)	29.72 (20.40 to 39.05)	19.39 (15.25 to 23.53)	18.61 (13.36 to 23.86)
	December	2.77 (2.42 to 3.13)	5.83 (4.55 to 7.12)	3.00 (2.59 to 3.40)	8.68 (5.85 to 11.51)	3.15 (2.18 to 4.12)	3.43 (1.84 to 5.03)

Average dairy cattle density was calculated across 2000, 2006, and 2014. Meshblocks were categorized as areas with no dairy, low dairy density (> 0–13.3 cows/km^2^), medium dairy density (> 13.3–102.3 cows/km^2^), and high dairy density (> 102.3 cows/km^2^). The categories of dairy cattle density were based on tertiles for all meshblocks with a dairy cattle density greater than zero.

**Figure 8:**
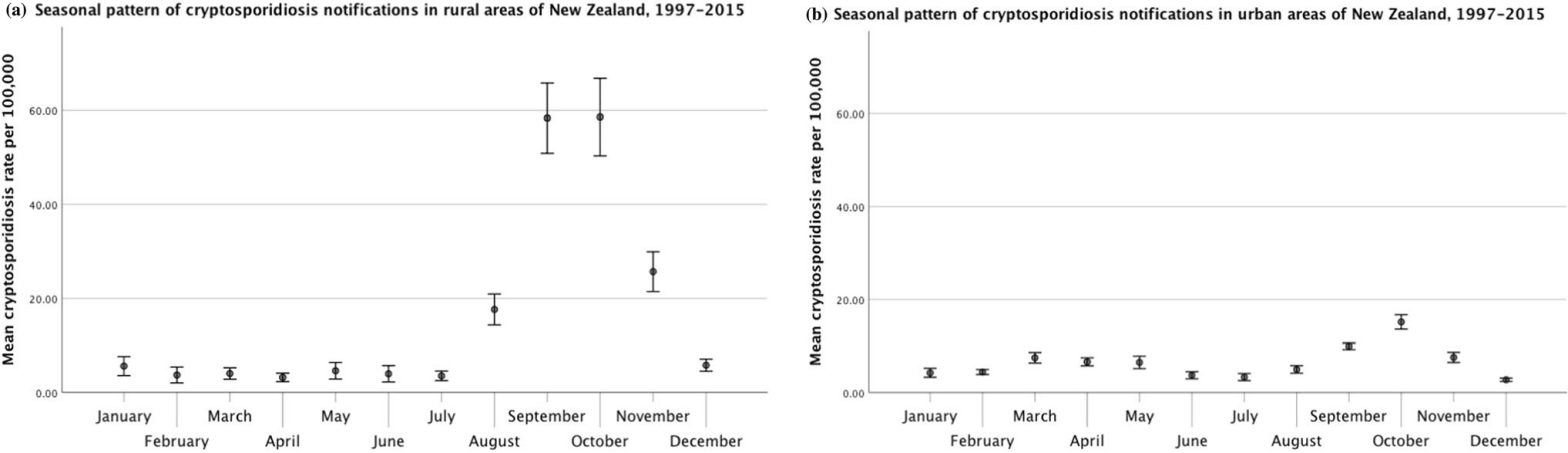
Seasonal pattern of human cryptosporidiosis rates stratified by (**a**) rural and (**b**) urban status. Error bars represent 95% confidence intervals for the means.

Cryptosporidiosis rates displayed a large spring peak in rural areas (Fig. 8, Table 1). Urban areas also displayed a small spring peak, as well as a very small autumn peak (i.e., March, April, and May) that was absent from rural areas.

Monthly plots were again created to examine the seasonality of disease notification rates for meshblocks with different average dairy cattle densities.

**Figure 9:**
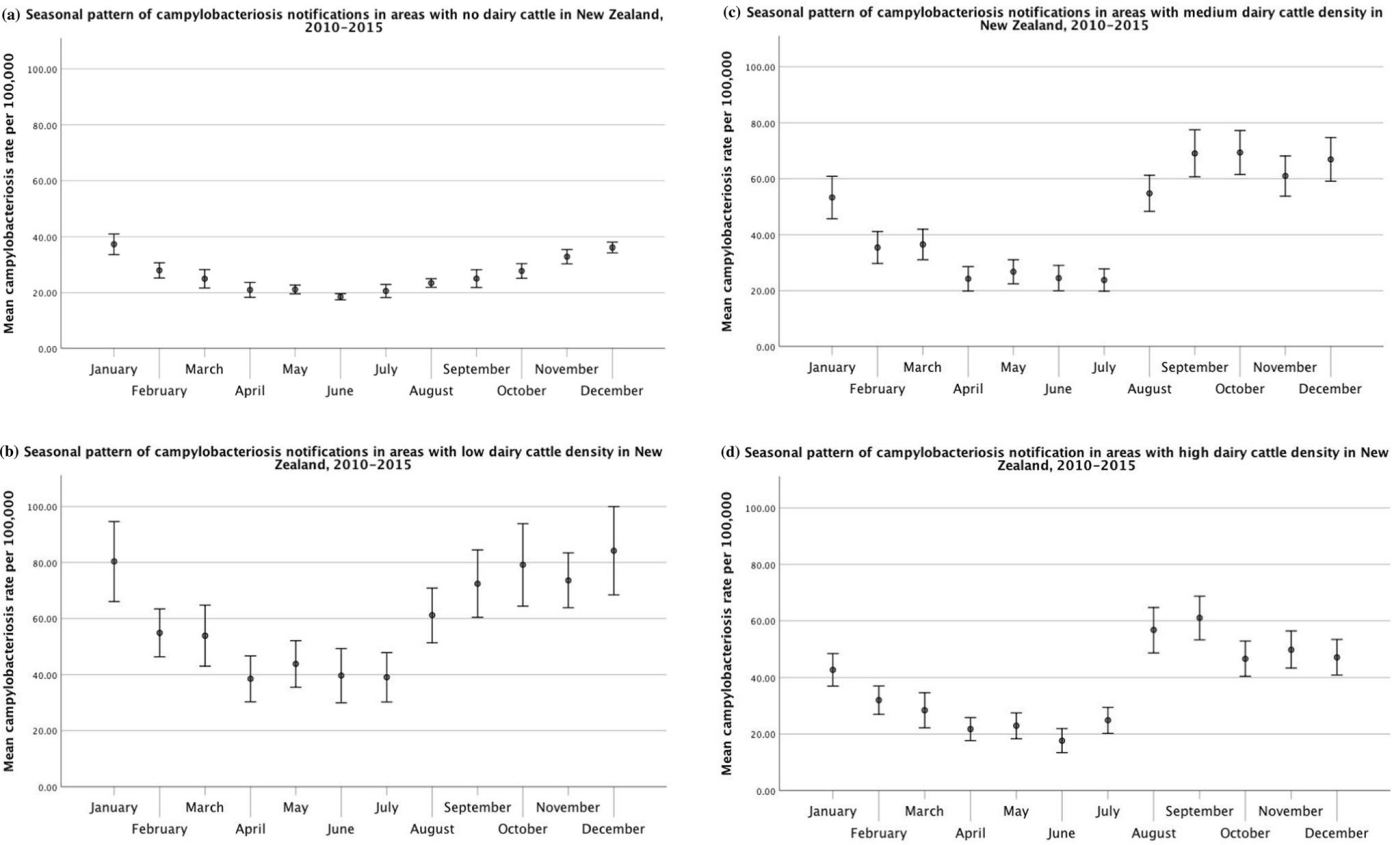
Seasonal pattern of human campylobacteriosis rates stratified by average dairy cattle density: (**a**) no dairy, (**b**) low dairy density, (**c**) medium dairy density, and (**d**) high dairy density. Error bars represent 95% confidence intervals for the means.

For campylobacteriosis, the plots below summarize the rates for each month from 2010 to 2015. Areas with no dairy cattle showed a small summer peak in campylobacteriosis rates, while areas with low, medium, and high dairy densities displayed a spring peak in addition to elevated summer rates (Fig. 9, Table 1). The spring peak was more distinct in areas with medium and high dairy densities. However, the highest mean rates were observed in areas with low dairy cattle density, albeit with wider confidence intervals due to greater year-to-year variability.

**Figure 10:**
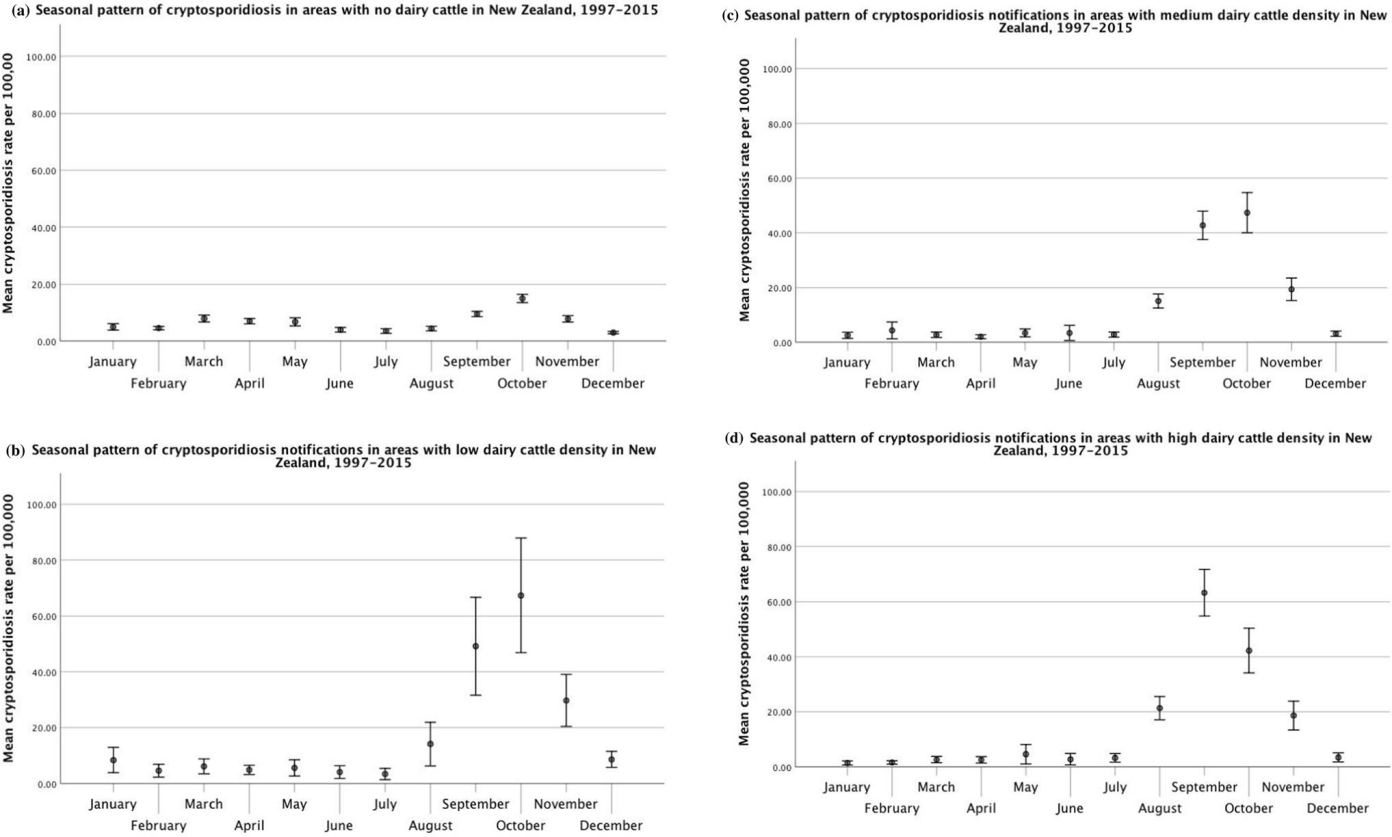
Seasonal pattern of human cryptosporidiosis rates stratified by average dairy cattle density: (**a**) no dairy, (**b**) low dairy density, (**c**) medium dairy density, and (**d**) high dairy density. Error bars represent 95% confidence intervals for the means.

In the case of cryptosporidiosis, all areas showed a marked spring peak (Fig. 10, Table 1). However, the spring peak in areas with no dairy cattle was much smaller than in areas with low, medium, or high dairy cattle density. Furthermore, areas with no dairy, and low and medium dairy density reached peak incidence in October, as opposed to areas with high dairy density, which reached peak incidence earlier (i.e., September). Meshblocks with no dairy cattle also displayed a slight increase in autumn that was absent from other areas.

## Discussion

Clear seasonal patterns of campylobacteriosis and cryptosporidiosis incidence were observed in this study, in line with previous research. There were distinct patterns in average monthly disease rates across urban and rural areas, during a period of greatly increased dairy intensification in rural New Zealand. Furthermore, to our knowledge, this study was one of the first to assess differences in disease incidence across areas with different livestock densities.

### Spatial Distribution of Dairy Cattle Density

The intensification of the New Zealand dairy sector has varied regionally, but maps of dairy cattle density indicate that most areas experienced increases in dairy cattle density from 2000 to 2014. The Waikato and Taranaki regions have long-established, major agricultural sectors (MacLeod and Moller 2006). Warmer winters and reliable rainfall have also allowed for the development of dairy farming in Northland, Bay of Plenty, Manawatu, Nelson, and the West Coast (Moran 1997; Smith and Montgomery 2004; MacLeod and Moller 2006). However, the dairy industry has also expanded into some regions that have not historically supported dairy cattle (MacLeod and Moller 2006). The Canterbury Plains, Otago, and Southland, which were previously considered unsuitable for dairy farming due to a lack of adequate rainfall and pasture, have seen the recent introduction and rapid growth of dairy farming (Smith and Montgomery 2004). These regions are largely reliant on irrigation to support their pastures (Smith and Montgomery 2004).

Additionally, a number of farms in New Zealand have transitioned away from other forms of agricultural production and into dairy farming (Smith and Montgomery 2004). There was a steady conversion of beef and sheep farms into dairy farms in the 1980s (Smith and Montgomery 2004; MacLeod and Moller 2006). It has been estimated that the area of dairy farming in New Zealand increased by 46% between 1972 and 1990 (MacLeod and Moller 2006). By 2016, New Zealand had an estimated 1.8 million hectares of dairy land (DairyNZ 2016). The maps of dairy cattle density generated for this study are potentially useful to researchers investigating the links between dairy cattle density and potential public health or environmental issues (e.g., other zoonotic diseases, nitrate contamination of drinking water resources, etc.) in New Zealand.

High dairy cattle density represents a significant potential exposure to pathogens. Cattle can excrete pathogens in their manure, and the pathogens can persist in the environment (FAO et al. 2006; Cavirani 2008; Toth et al. 2013; McDaniel et al. 2014). Cattle produce an average of 28 kg of manure (wet weight) each day (Vanderholm et al. 1984, Haynes and Williams 1993), the bulk of which goes untreated and is directly deposited to pasture (Bolan et al. 2009; FAO 2006; Ministry for the Environment 2016). Only a small proportion of dairy cattle excreta is collected and treated in New Zealand. It has been estimated that one dairy cow excretes fecal bacteria equivalent to that excreted by approximately 14 humans (Environment Waikato 2008; Foote et al. 2015), representing a nationwide equivalent of over 90 million people (Foote et al. 2015). However, the majority of human wastewater in New Zealand is treated to some extent before release (Taylor et al. 1997). The volume of untreated manure produced by New Zealand’s dairy cattle is of concern both for the environment and for public health.

### Temporal Patterns in Human Cases

Clear seasonal patterns were observed for human campylobacteriosis and cryptosporidiosis cases. Furthermore, there were notable differences between urban and rural areas, and between areas with and without dairy cattle. The seasonality of disease notifications can be explained by a number of different factors, such as climate, social and behavioral patterns, agricultural or environmental changes, and other drivers (Cherrie et al. 2018).

Weather has been linked to the seasonality of certain infectious diseases and can influence pathogen or vector survival and abundance, as well as host characteristics, such as behavior and susceptibility (Cherrie et al. 2018). In particular, research suggests that warmer temperatures could lead to increased pathogen proliferation and enhanced survival (Kovats et al. 2004; Fleury et al. 2006; Lal et al. 2012). This could in turn increase pathogen loads in animal reservoirs (Lal et al. 2012) and increase the length of transmission seasons (Semenza and Menne 2009; Lal et al. 2012).

The seasonal variation in the pathogen carriage rate in livestock, including dairy cattle, could also influence the seasonality of disease rates (Stanley and Jones 2003; Menrath et al. 2010; Williams et al. 2010). Evidence indicates that *Campylobacter jejuni* and *Cryptosporidium parvum* are widespread in newborn dairy calves in New Zealand (Grinberg et al. 2005; Al Mawly et al. 2015). Infected dairy calves may be a direct or indirect source of campylobacteriosis or cryptosporidiosis infection in humans (Grinberg et al. 2005; Snel et al. 2009), which may explain the seasonality of human disease rates associated with the spring calving period in New Zealand. Furthermore, certain farming management practices, such as grazing, overwintering, and herd movements, could influence pathogen shedding in cattle (Stanley and Jones 2003) and in turn influence human exposures and the seasonality of disease rates. The observed temporal patterns could also differ by pathogen strain (Learmonth et al. 2004; McCarthy et al. 2012; Friedrich et al. 2016), although strain specific data were unavailable for this study.

### Campylobacteriosis

Seasonal decomposition indicated that campylobacteriosis rates exhibit a distinct seasonal peak. The observed summer peak in campylobacteriosis may be due to a peak in *Campylobacter jejuni* clonal complex CC45. A time series study of genotyped human campylobacteriosis cases in Manawatu, New Zealand, from 2005 to 2013 found that CC45 was the only prevalent clonal complex to display a summer peak (Friedrich et al. 2016). However, CC45 is associated with a wide range of potential hosts and environmental sources (Levesque et al. 2008; Gripp et al. 2011; Sheppard et al. 2014; Friedrich et al. 2016).

In New Zealand, the literature has shown mixed results for the relationship between campylobacteriosis and climate (Lal et al. 2013), with both a time series analysis (Lal et al. 2013) and spatial analyses of campylobacteriosis determinants (Rind and Pearce 2010; Spencer et al. 2012) indicating that climatic factors were not significantly associated with long-term campylobacteriosis incidence in New Zealand. Furthermore, a review of the seasonality of campylobacteriosis in New Zealand and Europe indicated that the seasonal campylobacteriosis peak in New Zealand was more variable than in other countries (Nylen et al. 2002), which may indicate that there is a seasonal driver unrelated to climate (Lal et al. 2013). However, positive temporal associations between campylobacteriosis and temperature have been reported in a number of countries (Kovats et al. 2005), including the USA (Naumova et al. 2007) and the UK (Nichols et al. 2012; Abdelmajid et al. 2017; Cherrie et al. 2018). Campylobacteriosis was associated with temperature in the previous two weeks in a study in England and Wales (Nichols et al. 2012). A review of pathogen seasonality in England and Wales also indicated that *Campylobacter* had positive correlations with higher temperatures, sunshine, and vapor pressure in the previous month (Cherrie et al. 2018).

Box plots of average monthly campylobacteriosis rates displayed distinct patterns between urban and rural areas. Additionally, campylobacteriosis rates in rural areas were higher than in urban areas during most months. This finding suggests that there are different drivers of disease in urban versus rural areas, in line with previous research. For example, a campylobacteriosis source attribution study in New Zealand reported that poultry-associated cases were more likely to be reported in urban areas than in rural areas, whereas ruminant-associated cases were more likely to be reported in rural areas than in urban areas (Mullner et al. 2010). Ruminant strains were reported to pose a greater threat to children in rural areas in particular (Mullner et al. 2010). Box plots of average monthly campylobacteriosis rates by average dairy cattle density also showed a slightly different pattern between areas with no dairy cattle and areas with dairy cattle. Areas with dairy cattle displayed greater amplitude and higher seasonal peaks than areas without dairy cattle. Additionally, the spring peak occurred earlier in areas with medium or high dairy density than in areas with low density. The spring peak in rural areas and in areas with dairy cattle coincides with the calving season in New Zealand, and evidence indicates that *Campylobacter* spp. are widespread in newborn dairy calves and young cattle (Nielsen 2002; Grinberg et al. 2005). This pattern suggests that environmental exposure may play an important role in campylobacteriosis cases in rural areas.

### Cryptosporidiosis

Seasonal decomposition indicated that cryptosporidiosis rates exhibit a large spring peak in New Zealand. This finding is in line with several previous studies that reported a spring peak in cryptosporidiosis incidence in New Zealand (Lake et al. 2008; Snel et al. 2009; Lal et al. 2013). Box plots of average monthly cryptosporidiosis rates displayed distinct patterns between urban and rural areas, with a strong spring peak in rural areas and a smaller spring peak in urban areas. Urban areas also displayed a small peak in the autumn. The box plots also showed a distinct pattern between areas with no dairy cattle and areas with dairy cattle. Areas with dairy cattle displayed greater amplitude and higher seasonal peaks than areas without dairy cattle. However, areas with low dairy cattle density appeared to have higher seasonal peaks than areas with medium or high dairy cattle density, which may be due to the influence of other ruminants (i.e., sheep or beef cattle) or notification bias.

Evidence suggests that the autumn peak is predominantly *Cryptosporidium hominis* cases, while the spring peak is predominantly composed of *C. parvum* cases (Learmonth et al. 2004; Snel et al. 2009). Specifically, a study that examined *Cryptosporidium* oocysts isolated from human fecal specimens using the PCR-restriction fragment length polymorphism technique found that *C. hominis* was dominant in urban areas, while *C. parvum* was dominant in rural areas (Learmonth et al. 2004). Furthermore, a seasonal shift in transmission cycles was observed, with an anthroponotic cycle in the autumn and a zoonotic cycle in the spring (Learmonth et al. 2004; Knox et al. 2021).

In addition, monthly box plots indicated that the spring peak was earlier in rural areas than in urban areas. The spring peak was also earlier in areas with dairy cattle than in areas with no dairy cattle. This finding suggests that rural areas and areas with dairy cattle either have different drivers of disease, or that exposures in those areas may contribute to the burden of disease in urban areas and areas without dairy cattle. The spring peak is likely associated with agricultural practices, specifically the birth of newborn livestock (Learmonth et al. 2001; Snel et al. 2009). Calves younger than two months old are a major host for *C. parvum* (Grinberg et al. 2005; Al Mawly et al. 2015), but fewer than 1% of post-weaned calves and adult dairy cattle shed *C. parvum* oocysts in feces (Snel et al. 2009). Therefore, the spring peak may be due to calving (Snel et al. 2009).

Cryptosporidiosis has also been associated with weather and climatic conditions. In New Zealand, cryptosporidiosis has been associated with rainfall (Britton et al. 2010; Lal et al. 2013) and temperature (Lake et al. 2008; Britton et al. 2010; Lal et al. 2013). Specifically, a study that examined associations between regional climate variability and enteric disease incidence in New Zealand using Seasonal AutoRegressive Integrated Moving Average models found that the average temperature of the previous month was positively associated with cryptosporidiosis incidence (Lal et al. 2013). Similar findings have been reported in the US (Naumova et al. 2007), Australia (Hu et al. 2007), and the UK (Lake et al. 2005). A review of pathogen seasonality in England and Wales found that cryptosporidium had positive correlations with lower temperature variables (e.g., snow cover, ground frost, and air frost), precipitation, mean wind speed, and relative humidity in the previous month (Cherrie et al. 2018).

### Strengths and Limitations

A key strength of this study is the integration of different data sources to identify a potential public health issue that may be associated with the rapid intensification of dairy farming in New Zealand. However, there are limitations associated with the use of passive disease surveillance data to assess disease distribution, including underreporting, notification bias, and the misallocation of cases to geographic areas. Additionally, the patterns of disease described in this study could be strongly influenced by pathogen strain (Learmonth et al. 2004; Snel et al. 2009; McCarthy et al. 2012). However, strain-specific data were not available for this study. Additionally, population factors that influence disease risk (e.g., demographics, socioeconomic status, immunity status) were not accounted for in this study. While these factors are unlikely to change significantly over shorter time periods (e.g., monthly), they may vary on a longer timescale and could confound long-term temporal patterns.

## Conclusions

Campylobacteriosis and cryptosporidiosis rates displayed clear seasonal patterns. This finding was in line with previous research which has indicated that in temperate, higher-income nations, infectious enteric zoonoses exhibit seasonal patterns associated with weather conditions (Naumova 2006; Lal et al. 2012). This is also one of the first studies to assess differences in seasonality for urban versus rural areas, and for areas with different dairy cattle densities. Clear seasonal differences between disease notifications in urban and rural areas were apparent, suggesting that the determinants of disease differ for different sub-sections of the New Zealand population. Additionally, areas with dairy cattle had higher notification rates for both campylobacteriosis and cryptosporidiosis than areas that had no dairy cattle, with a peak in the spring that is likely due to increased exposure to cattle and fecal matter during the calving period. However, for both diseases, rates were highest in areas with low dairy cattle density, which may be due to the influence of other exposures or notification bias.

Results of this study support policies and practices to reduce and mitigate the environmental and public health harms of intensified agriculture in New Zealand. Specifically, given the links between animal and public health, interdisciplinary efforts are needed to monitor and control campylobacteriosis, cryptosporidiosis, and other zoonotic diseases. The promotion of behavioral changes in the human–animal–environment interface may help to reduce infection rates.

This study provides as starting point for additional research to determine the importance of livestock hosts, particularly dairy cattle, to human infections, and to ascertain the precise transmission pathways by which humans are exposed to zoonotic pathogens in New Zealand. Future research should also investigate the relationship between notified human cases, weather and climatic conditions, livestock management practices, environmental characteristics, and other variables that may help to explain seasonal patterns.

## References

[CR1] Abdelmajid D, Gordon N, Gianni L, Lora F, Anthony K, Sari K, Iain L, Christophe S, Richard E, Christopher L, Christophe H, Trevor B (2017). The seasonality and effects of temperature and rainfall on Campylobacter infections. International Journal of Population Data Science.

[CR2] Al Mawly J, Grinberg A, Prattley D, Moffat J, French N (2015). Prevalence of endemic enteropathogens of calves in New Zealand dairy farms. New Zealand Veterinary Journal.

[CR3] AsureQuality. (2019). "AgriBase." Retrieved 8 January 2019 from https://www.asurequality.com/our-solutions/agribase/.

[CR4] Bolan NS, Laurenson S, Luo J, Sukias J (2009). Integrated treatment of farm effluents in New Zealand’s dairy operations. Bioresource Technology.

[CR5] Britton E, Hales S, Venugopal K, Baker MG (2010). The impact of climate variability and change on cryptosporidiosis and giardiasis rates in New Zealand. Journal of Water and Health.

[CR6] Cavirani S (2008). Cattle industry and zoonotic risk. Veterinary Research Communications.

[CR7] Chen D, Moulin B, Wu J (2015). Analyzing and modeling spatial and temporal dynamics of infectious diseases.

[CR8] Cherrie MPC, Nichols G, Iacono GL, Sarran C, Hajat S, Fleming LE (2018). Pathogen seasonality and links with weather in England and Wales: a big data time series analysis. BMC Public Health.

[CR9] DairyNZ (2016). QuickStats about dairying-New Zealand. Retrieved 8 January 2019 from https://www.dairynz.co.nz/media/5418041/quickstats-new-zealand-2015-16.pdf2016.

[CR10] Dokumentov, A. (2018). Package stR. Retrieved 14 December 2021, from https://cran.r-project.org/web/packages/stR/vignettes/stRvignette.html.

[CR11] Environment Waikato (2008). The condition of rural water and soil in the Waikato region: risks and opportunities. Hamilton, N.Z., Environment Waikato.

[CR12] ESR (2017). Notifiable Diseases in New Zealand: Annual Report 2016. Porirua, New Zealand, Institute of Environmental Science and Research Limited (ESR).

[CR13] ESRI (2018). ArcGIS Desktop 10.5.1. Redlands, California, Environmental Systems Research Institute.

[CR14] FAO (2010). Greenhouse gas emissions from the dairy sector - a life cycle assessment. P. Gerber, T. Vellinga, C. Opio, B. Henderson and H. Steinfeld. Rome, Italy.

[CR15] FAO, H. Steinfeld, P. Gerber, T. Wassenaar, V. Castel, M. Rosales and C. de Haan (2006). Livestock's long shadow: environmental issues and options. Rome, Italy, Food and Agriculture Organization of the United Nations (FAO).

[CR16] Fleury M, Charron D, Holt J, Allen O, Maarouf A (2006). A time series analysis of the relationship of ambient temperature and common bacterial enteric infections in two Canadian provinces. International Journal of Biometeorology.

[CR17] Foote K, Joy M, Death R (2015). New Zealand dairy farming: Milking our environment for all its worth. Environmental Management.

[CR18] Friedrich A, Marshall JC, Biggs PJ, Midwinter AC, French NP (2016). Seasonality of Campylobacter jejuni isolates associated with human campylobacteriosis in the Manawatu region, New Zealand. Epidemiology and Infection.

[CR19] Grinberg A, Pomroy W, Weston J, Ayanegui-Alcerreca A, Knight D (2005). The occurrence of Cryptosporidium parvum, Campylobacter and Salmonella in newborn dairy calves in the Manawatu region of New Zealand. New Zealand Veterinary Journal.

[CR20] Gripp E, Hlahla D, Didelot X, Kops F, Maurischat S, Tedin K, Alter T, Ellerbroek L, Schreiber K, Schomburg D, Janssen T, Bartholomäus P, Hofreuter D, Woltemate S, Uhr M, Brenneke B, Grüning P, Gerlach G, Wieler L, Suerbaum S, Josenhans C (2011). Closely related Campylobacter jejuni strains from different sources reveal a generalist rather than a specialist lifestyle. BMC Genomics.

[CR21] Grout, L., M. G. Baker, N. French and S. Hales (2020). A Review of Potential Public Health Impacts Associated With the Global Dairy Sector. *GeoHealth* **4**(2): e2019GH000213 10.1029/2019GH000213 PMC7017588 32159049

[CR22] Haynes RJ, Williams PH (1993). Nutrient cycling and soil fertility in the grazed pasture ecosystem. Advances in Agronomy.

[CR23] Hu W, Tong S, Mengersen K, Connell D (2007). Weather variability and the incidence of cryptosporidiosis: Comparison of time series Poisson regression and SARIMA models. Annals of Epidemiology.

[CR24] Knox MA, Garcia-R JC, Ogbuigwe P, Pita A, Velathanthiri N, Hayman DTS (2021). Absence of Cryptosporidium hominis and dominance of zoonotic Cryptosporidium species in patients after Covid-19 restrictions in Auckland, New Zealand. Parasitology.

[CR25] Kovats RS, Edwards SJ, Charron D, Cowden J, D'Souza RM, Ebi KL, Gauci C, Gerner-Smidt P, Hajat S, Hales S, Hernandez Pezzi G, Kriz B, Kutsar K, McKeown P, Mellou K, Menne B, O'Brien S, van Pelt W, Schmid H (2005). Climate variability and campylobacter infection: an international study. International Journal of Biometeorology.

[CR26] Kovats RS, Edwards SJ, Hajat S, Armstrong BG, Ebi KL, Menne B (2004). The effect of temperature on food poisoning: a time-series analysis of salmonellosis in ten European countries. Epidemiology and Infection.

[CR27] Lake IR, Bentham G, Kovats RS, Nichols GL (2005). Effects of weather and river flow on cryptosporidiosis. Journal of Water and Health.

[CR28] Lake IR, Pearce J, Savill M (2008). The seasonality of human cryptosporidiosis in New Zealand. Epidemiology and Infection.

[CR29] Lake RJ, Campbell DM, Hathaway SC, Ashmore E, Cressey PJ, Horn BJ, Pirikahu S, Sherwood JM, Baker MG, Shoemack P, Benschop J, Marshall JC, Midwinter AC, Wilkinson DA, French NP (2021). Source attributed case-control study of campylobacteriosis in New Zealand. International Journal of Infectious Diseases.

[CR30] Lal, A. (2014). Evaluating the environmental and social determinants of enteric disease in New Zealand. Ph.D., University of Otago, Wellington

[CR31] Lal A, Dobbins T, Bagheri N, Baker MG, French NP, Hales S (2016). Cryptosporidiosis risk in New Zealand children under 5 years old is greatest in areas with high dairy cattle densities. EcoHealth.

[CR32] Lal A, Hales S, French N, Baker M (2012). Seasonality in human zoonotic enteric diseases: A systematic review. PLoS One.

[CR33] Lal A, Ikeda T, French N, Baker M, Hales S (2013). Climate variability, weather and enteric disease incidence in New Zealand: Time series analysis. PLoS One.

[CR34] Learmonth J, Ionas G, Pita A, Cowie R (2001). Seasonal shift in Cryptosporidium parvum transmission cycles in New Zealand. Journal of Eukaryotic Microbiology.

[CR35] Learmonth JJ, Ionas G, Ebbett KA, Kwan ES (2004). Genetic characterization and transmission cycles of Cryptosporidium species isolated from humans in New Zealand. Applied and Environmental Microbiology.

[CR36] Levesque S, Frost E, Arbeit RD, Michaud S (2008). Multilocus sequence Ttping of Campylobacter jejuni isolates from humans, chickens, raw milk, and environmental water in Quebec, Canada. Journal of Clinical Microbiology.

[CR37] LIC and DairyNZ (2019). New Zealand Dairy Statisitics 2018–19.

[CR38] MacLeod CJ, Moller H (2006). Intensification and diversification of New Zealand agriculture since 1960: An evaluation of current indicators of land use change. Agriculture, Ecosystems & Environment.

[CR39] McCarthy ND, Gillespie IA, Lawson AJ, Richardson J, Neal KR, Hawtin PR, Maiden MCJ, O'Brien SJ (2012). Molecular epidemiology of human Campylobacter jejuni shows association between seasonal and international patterns of disease. Epidemiology and Infection.

[CR40] McDaniel CJ, Cardwell DM, Moeller RB, Gray GC (2014). Humans and cattle: A review of bovine zoonoses. Vector Borne and Zoonotic Diseases.

[CR41] Menrath A, Wieler LH, Heidemanns K, Semmler T, Fruth A, Kemper N (2010). Shiga toxin producing Escherichia coli: identification of non-O157:H7-super-shedding cows and related risk factors. Gut Pathogens.

[CR42] Ministry for the Environment (2016). New Zealand's Greenhouse Gas Inventory: 1990–2014. Wellington, New Zealand

[CR43] Ministry of Health (2007). Direct Laboratory Notification of Communicable Diseases: National Guidelines. Wellington, New Zealand

[CR44] Moran W (1997). Farm size change in New Zealand. New Zealand Geographer.

[CR45] Mullner P, Jones G, Noble A, Spencer SEF, Hathaway S, French NP (2009). Source attribution of food-borne zoonoses in New Zealand: A modified Hald model. Risk Analysis.

[CR46] Mullner P, Shadbolt T, Collins-Emerson JM, Midwinter AC, Spencer SEF, Marshall J, Carter PE, Campbell DM, Wilson DJ, Hathaway S, Pirie R, French NP (2010). Molecular and spatial epidemiology of human campylobacteriosis: source association and genotype-related risk factors. Epidemiology and Infection.

[CR47] Naumova EN (2006). Mystery of seasonality: getting the rhythm of nature. Journal of Public Health Policy.

[CR48] Naumova EN, Jagai JS, Matyas B, DeMaria A, MacNeill IB, Griffiths JK (2007). Seasonality in six enterically transmitted diseases and ambient temperature. Epidemiology and Infection.

[CR49] Nichols GL, Richardson JF, Sheppard SK, Lane C, Sarran C (2012). Campylobacter epidemiology: a descriptive study reviewing 1 million cases in England and Wales between 1989 and 2011. British Medical Journal.

[CR50] Nielsen EM (2002). Occurrence and strain diversity of thermophilic campylobacters in cattle of different age groups in dairy herds. Letters in Applied Microbiology.

[CR51] Nylen G, Dunstan F, Palmer SR, Andersson Y, Bager F, Cowden J, Feierl G, Galloway Y, Kapperud G, Megraud F, Molbak K, Petersen LR, Ruutu P (2002). The seasonal distribution of campylobacter infection in nine European countries and New Zealand. Epidemiology and Infection.

[CR52] Patz J (2002). A human disease indicator for the effects of recent global climate change. Proceedings of the National Academy of Sciences of the United States of America.

[CR53] Rind E, Pearce J (2010). The spatial distribution of campylobacteriosis in New Zealand, 1997–2005. Epidemiology and Infection.

[CR54] Sears A, Baker MG, Wilson N, Marshall J, Muellner P, Campbell DM, Lake RJ, French NP (2011). Marked campylobacteriosis decline after interventions aimed at poultry, New Zealand. Emerging Infectious Diseases.

[CR55] Semenza JC, Menne B (2009). Climate change and infectious diseases in Europe. The Lancet Infectious Diseases.

[CR56] Sheppard SK, Cheng L, Méric G, De Haan CPA, Llarena AK, Marttinen P, Vidal A, Ridley A, Clifton-Hadley F, Connor TR, Strachan NJC, Forbes K, Colles FM, Jolley KA, Bentley SD, Maiden MCJ, Hänninen ML, Parkhill J, Hanage WP, Corander J (2014). Cryptic ecology among host generalist campylobacter jejuni in domestic animals. Molecular Ecology.

[CR57] Smith W, Montgomery H (2004). Revolution or evolution? New Zealand agriculture since 1984. GeoJournal.

[CR58] Snel SJ, Baker MG, Kamalesh V, French N, Learmonth J (2009). A tale of two parasites: the comparative epidemiology of cryptosporidiosis and giardiasis. Epidemiology and Infection.

[CR59] Spencer SE, Marshall J, Pirie R, Campbell D, Baker MG, French NP (2012). The spatial and temporal determinants of campylobacteriosis notifications in New Zealand, 2001–2007. Epidemiology and Infection.

[CR60] Stanley K, Jones K (2003). Cattle and sheep farms as reservoirs of Campylobacter. Journal of Applied Microbiology.

[CR61] Statistics New Zealand (2012). Dairy Industry 'mooooving' Forward. Web, Statistics New Zealand. Retrieved 8 January 2019 from http://www.stats.govt.nz/browse_for_stats/snapshots-of-nz/yearbook/environment/agriculture/dairy.aspx

[CR62] Statistics New Zealand. (2014). "Glossary and references." Standard for population terms Retrieved 8 January 2019 from http://archive.stats.govt.nz/browse_for_stats/population/standard-pop-terms/glossary-and-references.aspx#meshblock

[CR63] Statistics New Zealand (2017). Livestock numbers by regional council. New Zealand Statistics Agricultural Tables. New Zealand

[CR64] Taylor, R., I. Smith, P. Cochrane, B. Stephenson and N. Gibbs (1997). The state of New Zealand’s environment 1997. A. Saunders, D. Swain and B. Wall. Ministry for the Environment, Wellington, New Zealand

[CR65] Toth JD, Aceto HW, Rankin SC, Dou Z (2013). Short communication: Survey of animal-borne pathogens in the farm environment of 13 dairy operations. Journal of Dairy Science.

[CR66] Vanderholm, D. H., New Zealand Agricultural Engineering Institute, Pork Industry Council and Dairy Division of the Ministry of Agriculture and Fisheries (1984). Agricultural waste manual. Lincoln, New Zealand, New Zealand Agricultural Engineering Institute (NZAEI).

[CR67] Whitfield Y, Johnson K, Hobbs L, Middleton D, Dhar B, Vrbova L (2017). Descriptive study of enteric zoonoses in Ontario, Canada, from 2010–2012. BMC Public Health.

[CR68] Williams MS, Withee JL, Ebel ED, Bauer NE, Schlosser WD, Disney WT, Smith DR, Moxley RA (2010). Determining relationships between the seasonal occurrence of Escherichia coli O157:H7 in live cattle, ground beef, and humans. Foodborne Pathogens and Disease.

